# The postgraduate medical education pathway: an international comparison

**DOI:** 10.3205/zma001140

**Published:** 2017-11-15

**Authors:** Margot M. Weggemans, Bruce van Dijk, Birgit van Dooijeweert, Anne G. Veenendaal, Olle ten Cate

**Affiliations:** 1 University Medical Centre Utrecht, Centre for Research and Development of Education, Utrecht, The Netherlands; 2 University Medical Centre Utrecht, Utrecht, The Netherlands

**Keywords:** undergraduate medical education, postgraduate medical education, national licensing examinations, specialty training, residency program

## Abstract

An at first sight seemingly coherent, global medical workforce, with clearly recognizable specialities, subspecialties and primary care doctors, appears at a closer look quite variable. Even within the most progressive countries as to the development of medical education, with educators who regularly meet at conferences and share major journals about medical education, the differences in structures and regulations are big. This contribution focuses on the preparation, admission policy, duration, examinations, and national competency frameworks in postgraduate speciality training in Germany, the USA, Canada, the UK, Australia and the Netherlands. While general objectives for postgraduate training programs have not been very clear, only recently competency-frameworks, created in a limited number of countries, serve harmonize objectives. This process appears to be a challenge and the recent creation of milestones for the reporting on progress of individual trainees (in the US and in Canada in different ways) and the adoption of entrustable professional activities, a most recent concept that is quickly spreading internationally as a framework for teaching and assessing in the clinical workplace is an interesting and hopeful development, but time will tell whether true harmonization across countries will happen.

## Zusammenfassung

Was auf den ersten Blick als kohärente, globale Einheit der in der Medizin Beschäftigten erscheint, mit klar erkennbaren Fachgebieten, Teilfachgebieten und den Hausärzten, ist beim genaueren Hinsehen doch recht unterschiedlich. Auch in den in der Entwicklung der Medizinerausbildung fortschrittlichsten Ländern, in denen sich die Ausbilder regelmäßig auf Konferenzen treffen und die wichtigsten medizinischen Fachzeitschriften lesen, sind die strukturellen und gesetzlichen Unterschiede sehr groß. Dieser Beitrag beschäftigt sich mit der Vorbereitung, Zulassungspolitik, Dauer, den Examina und den nationalen Kompetenzrahmenbedingungen in der Facharztausbildung in Deutschland, USA, Kanada, dem Vereinigten Königreich, Australien und den Niederlanden. Die allgemeinen Ziele der Programme für die Postgraduiertenausbildung waren bisher nicht klar definiert, aber in der letzten Zeit werden übereinstimmende Ziele in Kompetenzrahmen gesetzt, die von einigen Ländern geschaffen wurden. Dieser Prozess stellt sich als schwierig heraus. Zwar wurden kürzlich Meilensteine für das Reporting von Fortschritten einzelner Auszubildender (in den USA und Kanada auf unterschiedliche Weise) geschaffen, und auch das vor Kurzem übernommene Konzept „entrustable professional activities“, das sich als Rahmen für das Unterrichten und Bewerten am klinischen Arbeitsplatz schnell international ausbreitet, scheint ein interessanter und hoffnungsvoller Ansatz, aber man wird erst mit der Zeit wissen, ob sich die Länder wirklich einander annähern.

## Introduction

For layman, there is no dispute concerning the definition of the concepts of “doctor” and “medical specialist”. Patients falling sick may attend these professionals anywhere in the world and expect to receive similar care. Behind the scenes however, there is much more disparity than many a layperson would think, both in the definition of what a doctor or specialist is and how to become one. 

Despite efforts of organisations like the European Union of Medical Specialists [https://www.uems.eu/], the pathway to becoming a medical specialist is indeed very different among countries worldwide [1]. Variation exists in admission policy, duration, terminology and significance of diplomas and licensing, and general structure of medical school and residency training. Confusion arises when similar terms refer to different stages of medical training, with different levels of competence and responsibility. 

One of the reasons why awareness of these differences in structure and terminology is important is the continuous increase in globalisation in healthcare, resulting in increasing numbers of migrating medical graduates and medical specialists [2], [3] with 25% of all physicians in the US, Canada and most West-European that are trained abroad [3]. This may lead to confusion when trained professionals must adapt to different medical systems and when countries do not mutually recognise each other’s medical degrees. The opposite may hold too. Within the European Union, legislation dictates that professional diplomas must be mutually recognized without further assessment [4], among countries that may show quite different career trajectories and educational objectives. EU’s assumption that all EU countries have similar training programmes is not based on thorough comparisons [5], [6]. Medical students and trainees, who probably have the best insight in details of programs, also take part in this migration as exchange students, and experience substantial differences in curricula and academic level [7], [8]. 

In 2013, Wijnen-Meijer et al. provided an overview of the structure and terminology of 40 different countries to address these issues [1]. The authors conclude that, even when countries mutually recognise diplomas, names of stages and degrees do not fully explain the education received and final level of training at graduation. Even the EU Bologna agreement, meant to harmonize all higher education, turned out to *increase* the disparity among medical schools in countries that signed the agreement as a minority introduced the required two-cycle model in medicine while most countries exempted medicine from this rule [9], [10]. 

Based on current literature, we conclude that little international comparison is available for postgraduate medical education whilst there are several reasons why this is relevant. The purpose of this contribution to the theme issue of Postgraduate Training therefore is to provide a more detailed overview of the different roads to specialty license for postgraduate medical students in a sample of six different countries. We used the definition of the World Federation for Medical Education (WFME) for postgraduate medical education, which reads “the phase in which doctors develop competencies under supervision towards independent practice after completion of their basic medical qualification, and might comprise pre-registration education (leading to right to independent practice), systematic vocational/professional education, specialist and sub-specialist education or other formalized education programmes for defined expert functions” [11]. For comparison purposes this terminology was applied to all six countries, even though we realize that in Australia the terms ‘pre-registration’ for primary medical education and ‘post-registration’ for junior doctor and specialist training are more common. 

## Methods

Six countries were identified for comparison of their postgraduate medical education structure. These include the United States of America, Canada, United Kingdom, Australia, the Netherlands, and Germany. The first 5 countries are in the forefront of medical education development and were chosen because of their known contribution to international medical education literature [12], [13], [14]. Germany was added since this article was meant as a contribution to the thematic edition “Postgraduate Medical Education” of the GMS Journal for Medical Education. Information on the duration of undergraduate and postgraduate education, existence of a national licensing examination, admission policy, and national competency framework for residency programs for each of the six countries was sought through a literature search. Additional information was found on the official website of relevant institutions, and in their official publications and documents. An earlier version of the article was sent to a medical education expert in each of the countries in order to check the facts stated on the situation in their respective countries.

## Results

Table 1 (Tab. 1) provides an overview of the main features of postgraduate medical education for each of the six countries. These features are described in more detail in the following sections. Table 2 (see attachment 1 ) contains an overview of all specialties that are offered in the six different countries. 

### Undergraduate medical education

Undergraduate medical education lasts 6 years in the Netherlands and Germany. In both countries students spend most of the second half of medical school on clinical rotations, in which they gain sufficient knowledge and skills to work as a junior doctor when they graduate [14], [15]. In Australia and the UK undergraduate medical education lasts 5 to 6 years [16], [17]. In the United States and Canada medical school lasts 4 years or, exceptionally, 3 years. In both countries, students are not usually eligible to start medical school directly after high school, but first need to obtain a 4-year bachelor’s degree (usually in the biomedical domain) [18], [19]. While non-American programs are most high-school entry, the Netherlands and the UK also offer 4-year graduate entry programs for medicine, for students who already obtained a degree in a different subject, but the number of places is limited [14], [20]. In Australia a combination of both high-school entry and college-entry medical schools can be found [17]. 

#### Postgraduate medical education and training

##### Internship

In Australia and the UK, medical school is followed by an internship that is obligatory for all medical graduates before entering postgraduate training. In Australia this period lasts 1 year and is called Postgraduate Year 1 (PGY1). In this year, interns do a series of rotations in which they gain experience in different healthcare environments. Satisfactory completion of this year is required for Australian junior doctors to be granted general medical registration by the Medical Board of Australia (MBA) [21]. 

In the United Kingdom, medical school graduates start a 2 year Foundation Program. In foundation year 1 training (F1) the transition from medical student to independent practitioner is made. Full registration is granted by the General Medical Council (GMC) after successful completion of F1. In foundation year 2 training (F2) junior doctors further develop core generic skills, take increasing responsibility for patient care, and begin to make management decisions. Successful completion of F2 is awarded with a foundation achievement of competence document (FACD), after which the foundation doctor can enter a specialty or general practice training programme [16], [22]. 

##### Work experience positions

In Australia, the Netherlands and the UK it is possible to gain clinical experience by working as a fully registered doctor before starting postgraduate training. In Australia most junior doctors who have completed PGY1 continue to work for at least one more year in public hospitals and community health services, because of a shortage of training places for most specialties and entry being highly competitive. In this time they usually rotate between clinical departments in regional and urban public hospitals, sometimes also including rural hospitals, community settings, and general practice. This provides junior doctors with experience in a broad range of clinical settings, while meeting health service needs at the same time [21]. 

In the Netherlands, full registration is granted upon graduation from medical school. The final year in most schools is a transitional year, in which students do a longer internship with increasing responsibility for patient care and other clinical activities. In this year they grow towards a level that resembles that of a starting resident, in order to ease the transition from undergraduate to graduate medical education [14], [23]. However, after graduation junior doctors can also opt to work as a resident-not-in-training to gain more work experience first. All medical graduates can apply for these resident-not-in-training employments directly at the hospital or institution where they would like to work. There is no formal training program for these pre-residency junior doctors, although in most hospitals they are able to attend scientific and educational activities for residents. 

In the UK, locum positions are offered when a temporary gap in specialty training programs occur. These positions can be filled by all registered doctors that completed the Foundation Program. There are two different kinds of locum appointments in the UK: Locum Appointment for Service (LAS) and Locum Appointment for Training (LAT). LAS are short term positions for a maximum of three months. Therefore little structured training can be offered and no accreditation of training can be granted. LAT positions last between three months and a year, and are granted training recognition that can contribute to obtaining the certificate of completion of specialist training (CCST). Locum positions are filled by foundation doctors after finishing the foundation program, for example to gain more time before making a choice for specialty training, but also by consultants, specialty doctors and general practitioners who prefer the flexibility these appointments offer [24], [25], [26]. 

##### National Licensing Examinations

In the Netherlands full registration is granted upon graduation from medical school. In Australia and the UK medical school graduates first need to complete one or two years of clinical work before they are fully registered. In Canada, the United States, and Germany passing medical licensing examinations are a necessary component for full registration and licensure.

The Medical Council of Canada Qualifying Examination (MCCQE) is one of the requirements for obtaining medical licensure in Canada. The MCCQE consists of two parts. MCCQE part I is a computer based test that is taken after completion of medical school. It assesses knowledge, skills and attitudes of medical graduates before they enter clinical practice in postgraduate training programs. MCCQE part II can be taken after a minimum of 12 months of postgraduate clinical medical training. This skills examination consists of clinical stations and is needed for medical licensure prior to independent clinical practice [27]. 

The United States have allopathic and osteopathic medical schools, which have their own licensure examinations. In order to obtain a license to practice medicine in the US doctors need to have completed medical school, passed the licensing examinations, and have completed at least one year of graduate medical education. Graduates from allopathic medical schools need to pass the United States Medical Licensing Examination (USMLE) [28], osteopathic graduates do the Comprehensive Osteopathic Medical Licensing Examination (COMLEX) [29]. Both USMLE and COMLEX consist of three parts, although USMLE step 2 is split up in two different parts: clinical knowledge and clinical skills. The first two parts of USMLE and COMLEX are completed during medical school, the third part at the end of the first year of graduate medical education. In many cases all three parts have to be completed within a seven-year period [18], [30]. 

In Germany, the national licensing examination is called state examination and consists of three parts. The first examination takes place at the end of the pre-clinical phase of undergraduate medical education. It is a written test (two days) and an oral examination (one day). The second state examination takes place before entry into the final (sixth) year and is an extensive written test for factual and clinical knowledge. It lasts three days. The third state examination takes place after the final year and includes extensive practical clinical parts as well as written tests for factual and clinical knowledge [15]. The completed third state examinations leads to full registration as a licensed physician. 

##### Admission policies for specialty training programmes

In the UK, the Netherlands, Australia, and Germany admission to a specialty training programme is obtained through an open, competitive selection. In Germany and the UK, all students that are granted full registration can apply for postgraduate training. In Australia, different specialties have different standards for the amount of postgraduate years of experience that is required from junior doctors before they can apply for the specific specialty training programme [21]. In the Netherlands, all medical school graduates can apply for specialty training with no fixed amount of clinical experience needed. However, since competition is high for many specialties, junior doctors with a few years of clinical experience, or for many specialties with research experience or even a PhD degree, are at an advantage [14]. 

The US and Canada use a matching system for applicants that want to obtain a place in postgraduate training programmes. The purpose of this matching system is to provide a uniform time for applicants and specialty training programmes to make their selections. In the US, the National Resident Matching Program (NRMP) [31] is the main match program, in Canada this is the Canadian Resident Matching Service (CaRMS) [32]. Applicants apply to 30-40 programs on average of their choice, do interviews at 12-15 programs and then make a rank order list of their preferences, which they submit to the match system. Residency program directors also submit their rank order list of applicants, after which the program matches programs and applicants. This Match Agreement is binding, which means that applicants must attend the program where they match [31], [32]. 

#### Specialty training per country: duration and competency frameworks

##### United Kingdom

In the United Kingdom, the General Medical Council (GMC) is responsible for setting standards for postgraduate medical education and training. Specialist training programmes in the UK last 3-8 years. There are two different types of specialist training. The first one is “run-through” training, where the trainee progresses to the next level automatically when all the required competences are sufficiently achieved. The “uncoupled” training programs are the second type and consist of 2-3 years of core training after which trainees have to go through another round of open competition in order to apply for a higher specialty training post. 

Postgraduate training is competence-based, with all specialty training programmes defining standards of knowledge, skills and behaviours according to the standards of the General Medical Council’s “Good Medical Practice”. In this document four domains are described:

1. knowledge, skills and performance;
2. safety and quality;
3. communication, partnership and teamwork; and
4. maintaining trust [33].

Assessment of progression of competence is achieved through formative assessment in the form of supervised learning events (SLEs), summative assessments of performance and examinations, and triangulated judgement by the educational supervisor. Satisfactory completion of a specialty training programme is awarded with a Certificate of Completion of Training (CCT), which enables entry to the specialist registers [34], [33]. 

##### Australia

In Australia, standards for postgraduate training are set by the Australian Medical Council, which acts as external accreditation body, but the management of training programmes differs between states and territories. Some training programmes are divided into basic and advanced components, starting with basic training which needs to be completed before progressing to advanced training. Many programs use both clinical and practical examinations and most have an exit exam [21]. Some training programs have competency-based elements, but this is not universal for the whole of Australia [35]. Training takes place in all public hospitals, private hospitals and community settings. The duration of the training programmes is 3-7 years. General Practice Training lasts 3-4 years, and consists of a combination of hospital rotations and general practice clinics [21]. 

##### The Netherlands

The College of Medical Specialisms (College Geneeskundige Specialismen, CGS) regulates specialty training and registration of medical specialists in the Netherlands. Most specialty trainings last 4-6 years, General Practice training lasts 3 years. Many specialty training programmes consist of time spent in both University Medical Centers (UMCs) and peripheral hospitals. A number of specialties require basic core training before starting (sub)specialty training. 

In the Netherlands, assessment during postgraduate medical education is competence based. All starting residents develop an “individual training plan” with the head of the training program, which is based on competencies that have already been obtained before the start of specialty training, for example during previous work as a resident-not-in-training. During specialty training, the resident works on a portfolio in which the progression on all competency domains is documented. The portfolio is the basis for progress evaluations for each resident [36], [37]. 

##### Germany

In Germany, specialty training lasts 5-6 years. Postgraduate training is not attached to academic centres, so that content consists almost entirely of work-based learning without formal taught courses [15], [38]. Postgraduate training in Germany is not yet competency-based, however it’s currently under reform and might become more competency-based in the coming years [39]. 

General practice training in Germany lasts 5 years, 3 of which consist of training in internal medicine in a hospital, of which 18 months may also be spent in ambulatory care including general practice. The other 2 years consist of training in general practice, of which 6 months may also be spent in surgery. In addition, 80 hours of training psychosomatic care are required as part of this training [38], [40]. 

##### Canada

In Canada, family medicine training lasts 2 years and is the shortest such residency in the world [41]. Other specialty training programmes are 4-6 years on average. The Canadian Medical Educational Directives for Specialists (CanMEDS) [42] framework describes all competencies physicians need to acquire in order to meet healthcare needs. The competencies are grouped into seven different roles, with “medical expert” being the integrating role, complemented by the roles of communicator, collaborator, leader, health advocate, scholar, and professional. To move towards competency-based medical education and enhance the assessment of whether competencies are actually acquired, the Royal College of Physicians and Surgeons of Canada (RCPSC) recently changed towards a model in which achievement of competencies is measured by milestones and Entrustable Professional Activities (EPAs). Milestones describe the expected progression of competence for each of the 7 CanMEDs roles throughout specialty training. EPAs are units of practice that can be entrusted to a resident once sufficient competence for this specific task has been shown. In one EPA, describing one specific task, multiple milestones and competencies can be integrated [43], [44]. 

##### United States of America

In the US specialty training programmes last 3-7 years. Family medicine training lasts 3 years. The Accreditation Council for Graduate Medical Education (ACGME) established 6 General Competencies (ACGME/ABMS General Competencies): patient care, medical knowledge, practice-based learning and improvement, interpersonal and communication skills, professionalism, and systems-based practice [45], [46]. The accreditation agency (ACGME) has, and takes, a powerful position to determine the requirements for all residency programs, that also semi-annually must report on developmental milestones for all speciality competencies. Next to the required competencies and milestones, EPAs are emerging in many postgraduate programs. The American Board of Pediatrics endorses EPAs on a national level [47], and other specialities are likely to follow.

## Discussion

This article aims to provide an overview of admission policies, duration, registration and licensing procedures in postgraduate medical training in six different countries. No two countries are alike on all aspects. One remarkable difference is the moment in time where full registration is obtained. In the Netherlands and Germany, full registration is granted upon graduation from medical school, which can be at age 24, or exceptionally even 23. In the UK, Australia, the US and Canada medical graduates must first work as junior doctors in an internship for 1-2 years or complete the first year of residency training before full registration is obtained. In Germany, the US and Canada passing medical licensing examinations are a necessary component for full registration and licensure.

The wealth of conceptual, practical and regulatory aspects of postgraduate medical education cannot be done justice fully within the space limitations of an article like ours. Most countries allow exceptions to general structures that could not all be addressed in this article. Our contribution was meant as a first comparison between six different countries and to give a flavour of existing differences. We believe it is important to communicate differences as migration and globalization require transparency. Exchanging comparisons may lead to mutual information and quality improvement.

## Acknowledgements

The authors would like to thank Dr Claire Touchie, Dr Gary Rogers, Dr Steven Durning, and Dr Richard Fuller for their comments on an earlier version of this manuscript and fact checking the information stated on the situation in Canada, Australia, the US, and the UK respectively. The authors however take full responsibility for any statements that may be incorrect.

## Competing interests

The authors declare that they have no competing interests. 

## Supplementary Material

(Sub)specialties offered in Australia, Canada, Germany, the Netherlands, UK, and USA*

## Figures and Tables

**Table 1:**
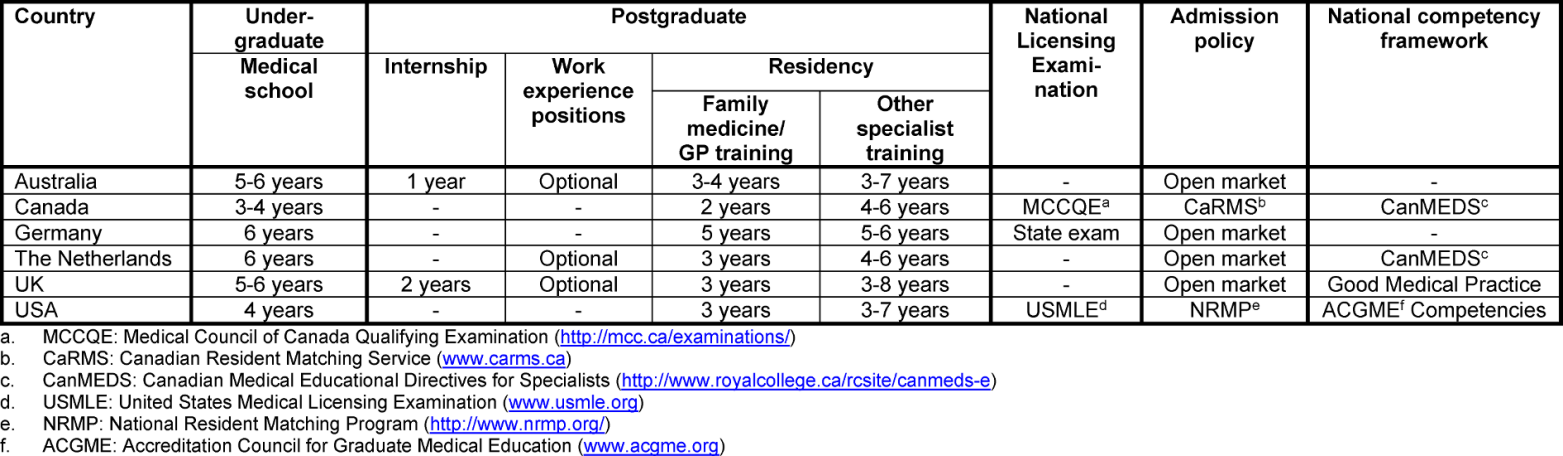
Selected features of postgraduate medical education in Australia, Canada, Germany, the Netherlands, UK, and USA
