# Supplementary material for: The postgraduate medical education pathway: an international comparison
Source: GMS J Med Educ. 2017 Nov 15;34(5):Doc63. doi: 10.3205/zma001140 (PMC5704606; doi:10.3205/zma001140)
Supplement: (Sub)specialties offered in Australia, Canada, Germany, the Netherlands, UK, and USA* [file JME-34-63-s-001.pdf]

(Sub)specialty	Australia[50]	Canada[52,53]	Germany[40]	Netherlands[49]	UK[48]	USA[51]
■ Specialty						
▒ Subspecialty						
Acute internal medicine				▒	■	
Addiction medicine	■			■		
Addiction psychiatry						▒
Adolescent medicine		▒				▒
Adult congenital heart disease						▒
Advanced heart failure and transplant cardiology						▒
Allergy (and immunology)	▒	▒		▒	■	■
Anatomical pathology	▒	■				
Anatomy			■			
Anesthesiology	■	■	■	■	■	■
Audio vestibular medicine					■	
Aviation and space medicine				■	■	■
Brain injury medicine						▒
Cardiac anesthesiology				▒		
Cardiac surgery		■	▒			
Cardiology (Cardiovascular disease)	▒	▒	▒	■	■	▒
(Cardio-)thoracic surgery	▒	▒	▒	■	■	■
Chemical pathology	▒				■	▒
Child abuse pediatrics						▒
Child and adolescent psychiatry		▒		▒	■	▒
Child mental health					▒	
Clinical biochemical genetics						■
Clinical cardiac electrophysiology						▒
Clinical cytogenetics and genomics						■
Clinical informatics						▒
Clinical molecular genetics and genomics						■
Clinical pharmacology and therapeutics (and toxicology)		▒			■	
Clinician investigator program		▒				
Colorectal surgery		▒				■
Community child health	▒				▒	
Community sexual and reproductive health					■	
Congenital cardiac surgery					▒	▒
Cosmetic dermatology				▒		
Critical care medicine		▒				▒
Cytopathology					▒	▒
Dermatology (and venereal diseases)	■	■	■	■	■	■
Dermato-oncology				▒		
Dermatopathology						▒
Developmental-behavioral pediatrics		▒				▒
Diagnostic ultrasound	▒					
Elderly care				■		
Emergency medicine	■	■		■	■	■
Endocrinology (and metabolism / diabetes mellitus)	▒	▒	▒	▒	■	▒
Epilepsy						▒
Family medicine / General practice	■	■	■	■	■	■
Female pelvic medicine and reconstructive surgery						▒
Forensic medicine			■	▒		
Forensic (histo)pathology	▒	▒			■	
Forensic psychiatry		▒	▒		■	▒
Gastro-enterology (and hepatology)	▒	▒	▒	■	■	▒
Gastro-intestinal surgery				▒		

General medicine						
Genetics (and genomics)						
Geriatric medicine						
Geriatric psychiatry						
Gynecologic oncology						
Gynecologic reproductive endocrinology and infertility						
Gynecological and obstetric ultrasound						
Gynecology and obstetrics						
Hand surgery						
Hematological pathology						
Hematology						
Hepatology						
Histopathology						
Hospital medicine						
Hygiene and environmental medicine						
Immunology						
Infectious diseases						
Insurance medicine						
Intellectual disability medicine						
Intensive care medicine						
Internal medicine						
Internal Medicine and Angiology						
Interventional cardiology						
Interventional radiology						
Laboratory medicine						
Liaison psychiatry						
Maternal and fetal medicine						
Medical administration						
Medical biochemical genetics						
Medical biochemistry						
Medical microbiology						
Medical physics						
Medical psychotherapy						
Medical virology						
Metabolic medicine						
Military medicine						
Molecular genetic pathology						
Neonatalogy (and perinatal medicine)						
Nephrology (Renal medicine)						
Neuro-anaesthesiology						
Neurodevelopmental disabilities						
Neurology						
Neuromuscular medicine						
Neuropathology						
Neuropediatrics						
Neurophysiology						
Neuroradiology						
Neurosurgery						
Neurotology						
Nuclear medicine						
Nuclear radiology						
Obstetric anesthesiology						
Occupational medicine						
Old age psychiatry						
Oncology						
Ophthalmology						
Oral and maxillofacial surgery						
Orthopedic sports medicine						
Orthopedic surgery (and trauma)						

Osteopathic neuromusculoskeletal medicine						
Oto(rhino)laryngology						
Pain medicine						
Palliative medicine						
Pathology						
Pediatric allergy, immunology (and infectious diseases)						
Pediatric anesthesiology						
Pediatric cardiology						
Pediatric clinical pharmacology and therapeutics						
Pediatric critical care medicine						
Pediatric dermatology						
Pediatric emergency medicine						
Pediatric (diabetes and) endocrinology						
Pediatric gastro-enterology and hepatology (and nutrition)						
Pediatric hematology (and oncology)						
Pediatric hospital medicine						
Pediatric infectious diseases						
Paediatric inherited metabolic medicine						
Pediatric intensive care medicine						
Pediatric nephrology						
Pediatric neurodisability						
Pediatric neurology						
Pediatric nuclear medicine						
Pediatric (medical) oncology						
Pediatric otolaryngology						
Pediatric palliative medicine						
Pediatric (and perinatal) pathology						
Pediatric (and juvenile) psychiatry and psychotherapy						
Pediatric radiology						
Pediatric rehabilitation medicine						
Pediatric respiratory (and sleep) medicine / Pediatric pulmonology						
Pediatric rheumatology						
Pediatric surgery						
Pediatric transplant hepatology						
Pediatric urology						
Pediatrics (and juvenile medicine)						
(Clinical) Pharmacology (Pharmaceutical medicine)						
Pharmacology and toxicology						
Phlebology						
Physiology						
Photodermatology						
Plastic surgery						
Plastic surgery within the head and neck						
Pre-hospital emergency medicine						
Proctology						
Psychiatry						
Psychiatry of learning disability						
Psychosomatic medicine (and psychotherapy)						
Public health (and preventive medicine)						
Radiotherapy (Radiation oncology)						
Radiology						
Rehabilitation medicine						
Rehabilitation psychiatry						
Respiratory medicine (Pneumology)						
Rheumatology						
Sexual health medicine						
Sleep medicine						
Special obstetrics and perinatal medicine						

Speech, voice and pediatric hearing disorders						
Spinal cord injury medicine						
Sport and exercise medicine						
Stroke medicine						
Substance misuse psychiatry						
(General) Surgery						
Surgical critical care						
Surgical dermatology						
Surgical oncology						
(Medical) Toxicology						
Transfusion medicine						
Transplantation medicine						
Transplant hepatology						
Tropical medicine (and international health)						
Undersea and hyperbaric medicine						
Urogynaecology						
Urology						
Genito-urinary medicine						
Vascular and interventional radiology						
Vascular medicine						
Vascular neurology						
Vascular surgery						
Visceral surgery						

* Some specialties have been clustered for comparison between the six different countries.

[40,48-53] Numbers refer to references. Please see reference list of main article for specific references.
